# An evidence appraisal of heart organoids in a dish and commensurability to human heart development in vivo

**DOI:** 10.1186/s12872-022-02543-7

**Published:** 2022-03-22

**Authors:** Dilip Thomas, Vinicio A. de Jesus Perez, Nazish Sayed

**Affiliations:** 1 Stanford Cardiovascular Institute, Stanford University School of Medicine, Stanford, CA 94305 USA; 2 Institute for Stem Cell Biology and Regenerative Medicine, Stanford University School of Medicine, Stanford, CA 94305 USA; 3 Division of Pulmonary and Critical Care Medicine, Department of Medicine, Stanford University School of Medicine, Stanford, CA 94305 USA; 4 Vera Moulton Wall Center for Pulmonary Vascular Disease, Stanford University School of Medicine, Stanford, CA 94305 USA; 5 Division of Vascular Surgery, Department of Surgery, Stanford University School of Medicine, Stanford, CA 94305 USA; 6 Stanford, CA USA

**Keywords:** Cardiac organoids, 3D platforms, Stem cells, Cellular crosstalk

## Abstract

Stem-cell derived in vitro cardiac models have provided profound insights into mechanisms in cardiac development and disease. Efficient differentiation of specific cardiac cell types from human pluripotent stem cells using a three-step Wnt signaling modulation has been one of the major discoveries that has enabled personalized cardiovascular disease modeling approaches. Generation of cardiac cell types follow key development stages during embryogenesis, they intuitively are excellent models to study cardiac tissue patterning in primitive cardiac structures. Here, we provide a brief overview of protocols that have laid the foundation for derivation of stem-cell derived three-dimensional cardiac models. Further this article highlights features and utility of the models to distinguish the advantages and trade-offs in modeling embryonic development and disease processes. Finally, we discuss the challenges in improving robustness in the current models and utilizing developmental principles to bring higher physiological relevance. In vitro human cardiac models are complimentary tools that allow mechanistic interrogation in a reductionist way. The unique advantage of utilizing patient specific stem cells and continued improvements in generating reliable organoid mimics of the heart will boost predictive power of these tools in basic and translational research.

## Background

Over the past decade, several strides made in stem cell research has led to unparallel opportunities to study human diseases in vitro. Cardiovascular diseases (CVD) comprising of congenital heart disease, heart failure, stroke and hypertension contribute toward nearly one-third of all deaths worldwide [1, 2]. Cardiomyopathies such as dilated cardiomyopathy (DCM), hypertrophic cardiomyopathy (HCM) and arrhythmogenic right ventricular cardiomyopathy (ARVC) have strong genetic basis. To study such diseases in addition to the ones caused by environmental, and pharmacological interventions, it is key to recapitulate fundamental functional characteristics of the heart. Both scarcity of clinically relevant samples and translational hurdles in extrapolating small animal-based studies notoriously impede scientific progress and create harmful misinformation [3]. Therefore, there is a need for advanced cardiovascular models that are three-dimensional, multicellular, functional, cost-effective and easy to manipulate. Cellular reprogramming techniques that efficiently wind the clock back on the cells to their embryonic-like stem cell state have been instrumental in derivation of different cell types of the heart. Particularly, directed differentiation of human induced pluripotent stem cell (iPSC)-derived cells have emerged as the major source for obtaining cardiac cells for various three-dimensional (3D) cardiac models. 3D models such as organoids, perfusable organ-on-a-chip models, and engineered heart tissues have been utilized for various disease modeling and restoration of heart function in vivo [4]. Despite the utility of engineered 3D cardiac models, stages involved in their derivation is strikingly different from in vivo. Cardiac morphogenesis in vivo is orchestrated in a cyclical manner through a series of cell expansion and intercellular signaling and differentiation of progenitor cell types. These processes also rely on electrical and mechanical maturation that induce local changes in shear stress, pressure and stretch due to blood flow. Developing methodologies to capture such dynamic interplay within in vitro cardiac organoids will accelerate our understanding of diseases by exploiting the potential of patient-specific iPSCs, to advance personalized medicine with further possibility of performing longitudinal population studies in a dish.

### Mapping cardiac morphogenesis and differentiation trajectories in vivo

The heart forms from the mesodermal embryonic tissue from anterior primitive streak. The cardiac specification and differentiation take place after the lateral movement of the anterior region into splanchnic mesoderm. This tissue further self-organizes into three distinct regions where the cardiac crescent or first heart field (FHF) is ‘sandwiched’ between head folds and second heart field (SHF). During the morphogenetic movements, the heart regions lie in close contact with pharyngeal or foregut endoderm [5]. Transcriptional activation of cardiomyogenesis is driven by inductive factors GATA4, TBX5 and SMARCD3 in the FHF. While the cardiac jelly secreted by the myocardial layer separates myocardial and endocardial layer, FHF transforms to linear structures of the primordial heart tube that gives rise to left ventricle after elongation and looping. Dorsal closure of the FHF derived hemi-tube next to foregut endoderm is completed around 18 days. At this stage, the sealed heart tube comprises of outer myocardial layer and inner endocardial layer. First contractions appear around between days 23 and 25, mechanical stimuli due to peristaltic pumping motion of the heart tube also contribute to chamber morphogenesis or ballooning [6]. Concomitantly, proliferative cells of SHF gives rise to arterial pole of the heart tube resulting in right ventricle, outflow tract myocardium and smooth muscle cluster posterior to great arteries. Venous pole which also originate from SHF give rise to atria, septa and inflow tract myocardium.

From a molecular point-of-view, orchestration of T-box containing transcription factors such as TBX20 and TBX5 trigger chamber and septa formation while BMP driven TBX2 and TBX3 driven activation leads to atrioventricular canal and outflow tract. Cardiac neural crest influx into the outflow tract and complete septation is completed around week 12 in humans where it resembles the heart structure morphologically [7]. Heart beats are regulated by the cardiac conduction system that originate at the venous pole. Concurrent transcriptional repression of NKX2-5 and activation of TBX18 and TBX3 lead to sinoatrial node differentiation. Together TBX5, TBX3 and NKX2-5 regulate morphogenesis of ventricular conduction system giving rise to trabecular structures from the endocardium. Endocardial- derived mesenchyme give rise to cardiac valves and vascular progenitor cells. A third layer called the epicardium emerge from a cluster of cells on the outer ventricular surface encapsulating the heart. Epicardium result in coronary smooth muscle cells and fibroblasts that play a key role in deposition of fibrous matrix and stimulating myocardial growth [8].This tissue-level coordination of differentiation and morphogenesis guided by extracellular matrix (ECM) is key to recapitulate embryonic cardiac development in a dish.

### Utilizing 3D cultures to mimic cardiomyogenesis: 2 decades of protocol refinement

In vitro differentiation of cardiomyocytes (CMs) from iPSCs follow sequential stages observed during embryonic development, wherein heart forming progenitor cells arise from the mesodermal cell layer sandwiched between the ectoderm and endoderm in the primitive streak. Cardiomyogenesis in vertebrates is driven by classes of growth factor proteins such as wingless/INT (WNTs), bone morphogenetic proteins (BMPs) and fibroblast growth factors (FGFs) [9, 10]. Concentrations and timely addition of activators or repressors of these pathways have resulted in directed differentiation methodologies to generate cells of cardiac lineage. Embryoid bodies (EBs) which are 3D clusters of pluripotent stem cells were first utilized to generate cardiomyocytes [11]. Further transition from 3D to 2D monolayer differentiation significantly improved the yield and differentiation efficiency due to increased bioavailability of signaling molecules [9]. One of the key advancements in in vitro recapitulation of cardiomyogenesis has been the identification of small molecules that overcome batch-to-batch variability and high-cost of growth factor-based approach [12]. Regardless of the method of derivation iPSC-CMs remain immature in terms of ultrastructure, electrophysiological properties, and metabolism. Several strategies have been developed to enhance maturation over the years using defined metabolic substrate cocktail, mechanical and electrical stimuli, and engineered extracellular scaffolding [13, 14]. A comprehensive overview of the traditional and current differentiation yield and maturation methodologies is described elsewhere [9, 15].

### Organoids for cardiac developmental disorders

**Fig. 1:**
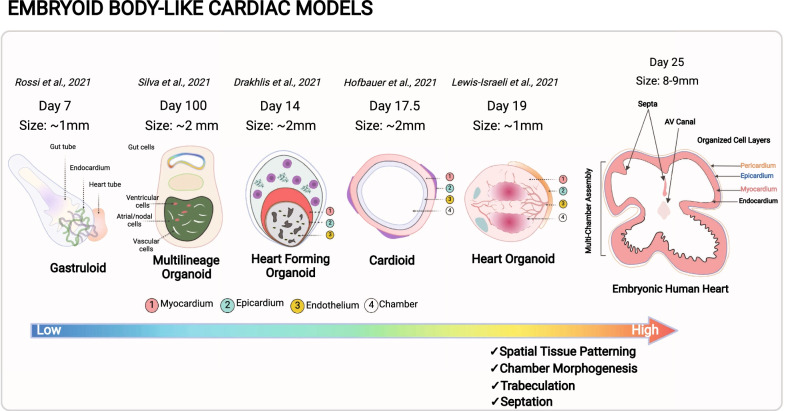
Embryoid body-like cardiac models. A schematic summary of feature-specific cardiac organoid models derived from human iPSCs. The figure highlights striking differences in in size, chamber and septation and tissue patterning between current cardiac organoid models and to its in vivo counterpart, thereby providing a premise for further innovation and optimization

Most recently, there has been a surge in cardiac ‘organoid’ models which are self-organized, spatially restricted clusters of cardiac-specific cell types derived from pluripotent stem cells. Some organoid models have been touted as new tools to study several congenital and developmental disorders such as hypoplastic left heart syndrome (HLHS) where the left ventricle fails to form due to mutation in NOTCH1 gene [16]. Several such mutations have been identified with developmental disorders such as atrial septal defect (ASD) associated with a mutation in GATA4 gene, and genes TBX5, TBX20 have been identified to result in ventricular septal defect (VSD) and left ventricular non-compaction (LVNC) [17]. With the use of human iPSCs and CRISPR technology, patient-derive cardiac cells carrying genetic mutations that lead to developmental disorders can be utilized to form cardiac organoids. However, given the primordial form of the cardiac organoids, and lack of commensurability to the scale and structure of heart during development, it is still challenging to form cardiac structures such as septa and heart valves using current organoid technologies. Methodologies to derive cardiac organoids and EBs are similar with minor modifications for cell-type specific enrichment. Improvement in our understanding of cardiogenesis, protocols and higher-resolution spatial imaging techniques has provided additional insight into intercellular cross-talk during early development. In addition to external cues, recent studies have shown a stronger cell fate determination within EB-like structures based on size and ECM mechanics. EBs of smaller sizes (150 µM diameter) favor higher endothelial cell fractions and larger EBs (400 µM diameter) give rise to more cardiac cell types due to differential expressing of WNT genes [18]. A higher control in manipulating reciprocal signaling between self-organized cell layers has the potential to shed light on developmental trajectory and defects that may occur during congenital development. The recent EB models that give rise to spherical structures (referred as ‘gastruloids’ [19] and ‘heart forming organoids’ [20]) resulting from WNT activation mimic cardiac morphogenesis partially with the formation of primitive gut-like structures that co-develop with fetal cardiomyocytes during early embryogenesis (Table 1). Gastrulation is an important embryonic hallmark; it is during this phase where mesodermal cells that gives rise to cardiac crescent and eventually cardiac tube. Impaired epithelial-to-mesenchymal transition in the endocardial region give rise to atrioventricular cushion and atrial septa defects. In gut forming organoids, cardiac progenitor populations and formation of a ‘tube-like’ structure can be observed, however it does not follow morphogenetic events that lead to segmentation of atrio-ventricular populations followed by looping to form four chambers. Hence these organoids maybe useful in tracing cell fractions that arise from FHF and SHF to predict restricted development due to mutation or other factors. Derivation of gastruloids and heart forming organoids utilize small molecules for WNT inhibition and activation instead of naturally occurring mesendoderm modulators and morphogens such as Activin A (ActA) and bone morphogenetic protein 4 (BMP4). In vivo, activation of WNT pathways occur in cyclical and region-specific manner which cannot be controlled while using small molecules that diffuse in culture disproportionately. A combination of small molecules and BMP4/ActA growth factors was shown to induce chamber-like cavity formation and enrichment of endothelial cell population [21]. A higher BMP4/ActA may also have resulted in higher atrial population in the heart organoids [22]. Hofbauer et al., demonstrated that with optimal ECM and WNT/BMP signaling conditions, single cavity forming early ventricle-like structures called ‘cardioids’ can be developed in the absence of foregut structures [16]. What makes ‘cardioids’ unique is cardiogenic induction through sequential activation of signaling pathways using BMP4/ActA, fibroblast growth factor (FGF), retinoic acid (RA), and WNT; wherein WNT dosage drives cavity morphogenesis and cardiomyocyte specification. Despite the introduction of newer terminologies for such feature-specific EBs, the resulting spheroid clusters lack of temporal and structural cues provided by other germ layers and additional lineages that form a four-chambered heart (Fig. 1). In a more recent example, a multilineage organoid protocol was introduced wherein there is a sequential emergence of cardiac mesoderm followed by gut endoderm over 100 days in culture [23]. The sequence of morphogenetic events reported in this organoid model is believed to recapitulate stages that occur after the ‘heart tube’ formation. Quite interestingly compared to the other organoids, this model supports preferential differentiation of atrial and nodal cells over ventricular cardiomyocytes. In contrast to in vivo cardiac patterning, it is clear that the organoid models are dissimilar in cardiac chamber morphogenesis, diversity in cell populations and presence of definitive conduction system that coordinates heartbeat. There are several contributing factors that limit current approaches to faithfully generate cardiac organoids. One of the key factors is the dissimilar WNT modulation strategy with small molecules that do not precisely target primed primitive streak, and further temporally activate gene programs in a region-specific manner. Secondly, it is necessary for all cardiac stem cell lineages to co-exist in a structure similar to a ‘heart tube’ as they produce local morphogens and help tissue expansion to build higher levels of structural complexity guided by cell polarity and ECM. Lastly, control over mechanical stresses and cellular composition holds the key for 4-chambered patterning and septation with initiation of beating by the cardiac conduction system at week 3 contrary to early beating observed in vitro in the absence of heart tube formation. Hence, it is important to leverage new experimental approaches that capture molecular switches to more precisely recapitulate the dynamics of heart development, both in structure and composition. In the meanwhile, one should carefully evaluate the limitations of the current organoid models and build a strong rationale for experimental endpoints to obtain meaningful interpretations. These endpoints must include both short-term and long-term culture duration to not only understand the impact on maturation, but also fidelity of cellular responses as a function of time-dependent remodeling.

**Table 1:** A comparison of embryonic heart organoid protocols and features

Organoid protocol	WNT activators/growth factors	Differentiation/Maintenance period	Cell population markers	Embryonic morphology	Cardiac maturation features	References
Cardioids	8–30 ng/mL FGF2, 5 µM LY294002 (PI3K inhibitor), 50 ng/mL activin A, 10 ng/mL BMP4, 10 µg/mL insulin, 4–9 µM CHIR99021 and 5 µM IWP2	7.5 days/3 months	FHF markers (HAND1, TBX5, NKX2-5), ventricular (IRX4, MYL2), atrial (NR2F2, HEY1); Intermediate WNT inhibition + VEGF resulted in 41% atrial (MYL7) and 53% endothelial (CDH5) cell fractions and EC-derived fibroblasts (SOX9, MSX1/2, COL1A1, and COL3A1)	Concentric layers of epicardium, myocardium and endothelium lined cavity	β-adrenergic receptors 1 and 2, higher expression of HERG, KCNH2, TNNI1, TTN, MYH6, RYR2 and ATP2A2	[16]
Gastruloids	3 µM Chi99021, 30 ng/mL bFGF, 5 ng/mL VEGF	7 days	Mesp1, FHF markers (NKX2-5, TBX5, and HCN4), SHF markers (TBX1, ISL1, and FGF10), Endothelial (FLK1) and Cardiac (cTnT)	Cardiac crescent-like domain, endocardial-like layer, gut-like tube	ACTN2 and RYR2	[19]
Heart forming organoids	7.5 µM CHIR99021, 5 µM IWP2	10 days/4.7 months	Cardiac (NKX2-5, TNNT2), Endothelial (CD31, CDH5), Lung (SOX2, SOX17,) Pharyngeal endoderm (ISL1, PAX9, TBX1), Liver (HNF4α, AFP)	Cardiac mesoderm, proepicardium	–	[20]
Heart organoids	2–4 µM CHIR99021, 1.25 ng/mL BMP4, 1 ng/mL activin A, 2 µM C59	15 days	FHF (HAND1), SHF (HAND2), epicardial marker (WT1, TJP1); 59% cardiac (TNNT2), 16%, 14% endocardial (NFATC1), 12% fibroblast (THY1), 1.6% endothelial (PECAM1). ~ 48% atrial (MYL7), ~ 18% ventricular (MYL2)	Epicardium, endocardium, myocardium and multiple chambers	–	[21]
Cardiac and gut (multilineage) organoid	12 µM CHIR99021, 5 µM IWP2,10 µM ROCKi	30 days/11.8 months	Day 20 Cardiac (cTnT), ventricular (MLC2v), endothelial (PECAM1), epicardial (TBX18); Day 30 hindgut (FOXA2, CDX2); Day 60 smooth muscle (aSMA) 80% atrial/nodal:20% ventricular, Day 70–100 paneth, goblet cells and > 90% atrial/nodal cells	Gut endoderm, subepicardium, and cardiac mesoderm	High amplitude and stroke velocity at 1 Hz with response to stimulation at 8 Hz after 80 days, > 70% cells with aligned sarcomeres with ~ 4 aspect ratio	[23]

### Modular approaches to dissect cellular crosstalk in cardiac diseases and drug screening

Developmental cardiac organoid-like models do not serve as an attractive tool to perform investigations in a reductionist manner due to lack of high fidelity in cellular composition and niche. Diseases where the pathophysiology is unclear in vivo due to complex microenvironment could benefit from engineered tissue-like structures that can be manipulated in a modular fashion. Several protocols have been developed in recent years to generate major non-myocyte cell types of the heart from iPSCs such as endothelial cells [24], cardiac fibroblasts [25], smooth muscle cells [26] and macrophages [27]. Feasibility in generating these cell types provides an “off-the-shelf” approach to build both cellular complexity and tailor extracellular microenvironment. Assembly of cell and microenvironment using tissue engineering can be accomplished using several different methods. Cardiac cell types embedded in an artificial ECM can be trained with mechanical or electromechanical stimulus and exhibit anisotropy such as ‘engineered heart muscle (EHM)’ [28, 29], ‘cardiac muscle thin-film’ [30] and ‘cardiac wires’[31]. ECM-free forced aggregates in form of isotropic ‘cardiac spheroids’ offer higher throughput and ease of fabrication to dissect cell–cell interactions independent of a guiding ECM [32]. Both these types of models deem useful to unravel contractile and electrophysiological deficits that occur due to inherited mutations, drug sensitivities or exposure to environmental chemicals. From a disease modeling perspective, these models can be employed to capture the influence of multiple cell types and how each cell type contributes toward a clinical phenotype [24]. These models are also amenable to CRISPR technology to understand the mechanistic influence of genes in development and disease with genome-wide CRISPR screens and generation of reporter lines [33]. However, these miniature models do not allow recapitulation of volumetric and pressure outputs with clinically relevant cardiac performance measures. A more sophisticated scaffolding, which can be fabricated using bioprinting or electrospun fibers is needed to match the shape, size, and mechanical strength of the heart [34].In such models, pressure–volume dynamics serve as an important parameter where typical systolic values at mid-embryonic state is ~ 2 mmHg and ~ 60–90 mmHg at neonatal stage. To date among the tissue-engineered chamber models a low pressure of 0.23 mmHg has been achieved [35]. Patterning vascularization networks for nutrient and drug diffusion has been one of the major hurdles to enable long-term maintenance and maturation of engineered systems. Heart-on-a-chip devices that are typically the size of a credit card consisting of compartments for tissue assembly, interconnected via channels has been a vital upgrade in mimicking nutrient flow to the tissue. These devices referred as microphysiological systems (MPS) do not aim to mimic the function at an organ-scale but are useful in modeling fundamental tissue-scale responses to external stressors or compounds. MPS combines existing scaled-down cardiac models, biomechanical forces, and fluid-flow to allow real-time assessment of changes in function with the aid of biosensors [36]. MPS platforms also allow organ multiplexing for precise interrogation of drugs and the effect of secondary metabolites shuttling between other tissues such as lung, liver or even tumors [37, 38]. Such systems have an important translational value as a ‘preliminary screen’ with a well-defined context of use, and realistic pharmacokinetic/pharmacodynamic endpoints.

### Tempering expectations and addressing current challenges in 3D cardiac models

Both cardiac organoid and heart-on-a chip models are excellent surrogates to understand several important aspects of heart function during development and disease. However, it is important to acknowledge that utility in predicting in vivo functions will remain limited due to stochastic self-patterning variability between every batch of stem cell differentiation. In addition, several factors that currently rely on self-assembly time need to be coupled with controlled delivery of cues. For example, in addition to progenitor-cell types, cardiogenesis is driven by a combination of long- and short-range paracrine interactions, gene regulatory networks and ECM-guided geometrical cues. Efforts toward obtaining blueprints of stage-wise development will enable us to isolate and embed temporal cues in these models to influence multi-axial patterning and cellular organization, rather than focusing on autonomous self-assembly under different conditions. Another important aspect in cardiac models is obtaining innervation and vascular anastomoses which provide feedback on carbon dioxide, dissolved oxygen and fluid pressure. Technical challenges that prevent widespread use of cardiac models can be addressed through standardized characterization of stem cell sources, using well-defined platforms and most importantly choosing the right model in the context of use [36]. With increasing cellular complexity within 3D tissue models, multiplexing tissue systems on a chip and incorporation of elements of immune system would require an inevitable investment in development of bespoke medias [39]. Such efforts would exponentially improve reproducibility of measurements given physiological buffered state for cellular and tissue homeostasis. Furthermore, introduction of multicellular cardiac organoids integrated into biosensor laden multiorgan systems-on-a-chip will help in improving reliability through remote measurement of organ level functions and complex metabolic interactions.

## Conclusion

Currently, both ECM and ECM-free cardiac models are proving useful as an obligate substitute for pre-clinical animal models, to validate clinical observations and as a screening tool for therapeutics or toxicology. With success through continued efforts in addressing some of the discussed challenges, we must revisit our expectations and utility of the model based on evidence and reproducibility. For example, current 2D and 3D tissue engineered models with limited heart-like features would be useful from a reductionist approach to understand cell specific mechanism or direct pharmacological response. Future availability of morphologically and spatially defined organoid mimics will further assist in mapping direct and indirect interactions due to concerted cellular response and fluidic stress. Such systematic approach toward building complexity will drive utility-based research in cardiovascular organoid biology as a complimentary tool and not a panacea for studying multifactorial cardiovascular diseases.

## Data Availability

Not available.
